# Picolinic Acid in Patients with Chronic Hepatitis C Infection: A Preliminary Report

**DOI:** 10.1155/2012/762863

**Published:** 2012-04-10

**Authors:** Jolanta Zuwała-Jagiello, Monika Pazgan-Simon, Krzysztof Simon, Maria Warwas

**Affiliations:** ^1^Department of Pharmaceutical Biochemistry, Wroclaw Medical University, Poland; ^2^Clinic of Infectious Diseases, Liver Diseases and Acquired Immune Deficiency, Wroclaw Medical University, Poland

## Abstract

Macrophage activation seems to be a feature of chronic liver diseases. Picolinic acid (PA) as a macrophage secondary signal causes the activation of interferon-gamma- (IFN-*γ*-) prime macrophage and triggers cytokine-driven inflammatory reactions. The rationale for seeking increased PA formation in chronic viral hepatitis is based on the involvement of activated macrophages in chronic viral hepatitis-associated inflammation. The aim of this study was to determine serum PA levels in patients with chronic hepatitis C infection, taking into account the presence of diabetes. We assessed PA and high-sensitivity C-reactive protein (hsCRP) as a marker of inflammation in 51 patients with chronic hepatitis C infection (CHC), both with and without diabetes and 40 controls. Compared with the controls, the patients with CHC showed a significant increase in plasma concentrations of PA and hsCRP (*P* < 0.01 and *P* < 0.05, resp.). The values of PA and hsCRP were more elevated in patients with diabetes than without diabetes (both *P* < 0.01). The positive relationships were between PA and hsCRP levels (*P* < 0.05) and the presence of diabetes (*P* < 0.001). We documented that significant elevation in serum PA levels is associated with diabetes prevalence and increased inflammatory response reflected in hsCRP levels in CHC patients.

## 1. Introduction

Hepatitis C virus (HCV) infection has been associated with various extrahepatic manifestations, and diabetes mellitus is one of these. Diabetes in patients with chronic hepatitis C infection (CHC) has unique and complex pathogenesis, which distinguishes this metabolic disorder from type 2 diabetes mellitus. In this regard, a high prevalence of diabetes has been reported in HCV-infected patients in comparison with other liver diseases [1–3]. Mehta et al. [4] have reported that preexisting HCV infection may increase the risk of type 2 diabetes mellitus in patients with recognized diabetes risk factors. The mechanisms by which chronic hepatitis C infection induces increased insulin resistance and the risk for development of diabetes have not been completely clarified. It has been observed that type 2 diabetes mellitus occurs in the early stages of liver disease [5]. In the light of these observations, the mechanism through which chronic hepatitis C infection is associated with insulin resistance may involve direct viral effects, proinflammatory cytokines, and suppressors of cytokine signaling (reviewed in [6]). 

In particular, inflammation has been hypothesized to underlie not only the pathogenesis of the diabetes but also its association with cardiovascular diseases [7–12]. The prototypic marker of inflammation is C-reactive protein (CRP). Numerous studies, especially in normal individuals, have shown that CRP levels in the highest quantile predict cardiovascular events [11, 12]. Similarly, both white blood cell (WBC) count and erythrocyte sedimentation rate (ESR) have been associated with cardiovascular risk [13, 14]. There is also evidence supporting the suggestion that CRP levels are increased in diabetes [7, 15, 16]. Moreover, accumulated evidence suggests that HCV increases the risk of atherosclerotic cardiovascular disease by causing insulin resistance, diabetes, and inflammation [3].

Tryptophan (TRP) exceeding basal requirement for protein synthesis is oxidized *via* indole-ring cleavage through the kynurenine pathway, consisting of several enzymatic reactions leading to 2-amino 3-carboxymuconate 6-semialdehyde (ACMS) [17]. ACMS can be decarboxylated to 2-aminomuconate 6-semialdehyde (AMS) by the enzyme ACMS decarboxylase (ACMSD), or it can undergo spontaneous pyridine ring closure to form quinolinate, an essential precursor for *de novo* NAD synthesis. AMS can be converted nonenzymatically to picolinic acid (PA) or routed to the citric acid cycle *via* the glutarate pathway. TRP catabolism, initiated by indoleamine 2,3-dioxygenase (IDO), leads to the production of biologically active molecules. Interferon-*γ* has been shown to be a potent stimulating cytokine for IDO *in vitro*. Similarly *in vivo*, a decrease in serum tryptophan and an increase in kynurenine in parallel were exhibited, indicating an enhanced degradation of tryptophan, while the cellular immune system is activated. In particular, the ratio of kynurenine to tryptophan (KYN/TRP) seems to be a sensitive indicator for interferon-*γ*-induced tryptophan degradation (reviewed in [18]).

The role of the tryptophan, particularly during inflammation, is just beginning to be appreciated, and a great deal of recent research has focused on the effects of tryptophan catabolites on effectors cell function [19]. TRP catabolites, biologically active molecules [20], represent a relatively newly identified class of stimuli, which differ from classical immunological stimuli because of their capability to signal to the macrophages through mechanisms that do not require a cell-surface receptor. Among them, picolinic acid (PA), a terminal metabolite of tryptophan degradation detected *in vivo* in human sepsis [21] and in the serum of patients with liver cirrhosis [22], is capable of triggering mouse macrophages production of a number of inflammatory gene products, such as reactive nitrogen intermediates [23] and cytokine [24]. Furthermore, TRP-derived catabolites have been detected *in vivo* in patients with viral infections, malignant and inflammatory diseases [25–27].

The results of numerous studies strongly suggest that macrophage activation seems to be a feature of chronic liver diseases [28–30]. However, other studies have suggested a role for TRP-derived catabolizes in macrophages activation [21, 23]. The rationale for seeking increased PA formation in chronic viral hepatitis is based on the involvement of activated macrophages in chronic viral hepatitis-associated inflammation. Elevated formation of PA in patients with chronic hepatitis C infection might be expected due to enhanced TRP breakdown demonstrated in inflammatory reactions. The aim of this study was to determine serum PA levels in patients with chronic hepatitis C infection, taking into account the presence of diabetes.

## 2. Patients and Methods

### 2.1. Patients and Study Design

This study was performed in 51 patients with chronic hepatitis C infection (CHC) admitted to the Clinic of Infectious Diseases, Liver Diseases and Acquired Immune Deficiency for evaluation. The control group consisted of healthy blood donors with normal aminotransferases, normal blood counts, and negative markers for virus hepatitis and HIV (23 males/17 females, median age 32.5 years, range 19–65 years). No statistically significant difference between the CHC patients and healthy controls in female/male ratio (**P** = 0.51) was found. There was a significant difference in age, but PA level was not age related in neither the controls (**r** = 0.14, **P** < 0.21) nor the CHC patients (**r** = 0.17, **P** < 0.34). Clinical and biochemical characteristics of the study group are reported in detail in Table 1.

 The exclusion criteria were as follows: (1) liver cirrhosis; (2) conditions other than diabetes and HCV infection that could influence either serum glucose levels: premenopausal women; alcohol consumption >40 g/day; treatment with steroid or no steroidal anti-inflammatory drugs, except aspirin; concomitant infection; chronic diseases other than diabetes; (3) type 1 diabetes (history of diabetic ketoacidosis or age <30 years with insulin requirement) and secondary diabetes due to chronic pancreatitis or pancreatic tumor.

 Liver cirrhosis was ruled out by liver biopsy performed within 18 months before inclusion (compensated patients) or by typical clinical features such as signs of portal hypertension (splenomegaly, ascites, and esophageal varies), hematologic evidence of hypersplenism, or biochemical evidence of hepatocellular failure.

 The diagnosis of chronic hepatitis C infection was based on persistently increased alanine aminotransferase values, anti-HCV and HCV-RNA positivity, and liver histology features. The HCV inflammation was confirmed by measurement of HCV Ab and HCV RNA in the serum, using the EIA methods and RT PCR-Cobas Amplicor Roche methods, respectively. For the anti-HCV-positive patients with normal aminotransferase levels and no liver biopsy, we ensured that aminotransferase levels and liver function tests results were persistently normal.

 Patients were divided in two groups according to their HCV antibody status and the presence of diabetes: anti-HCV-positive diabetic patients (*n* = 22) and anti-HCV-positive nondiabetic patients (*n* = 29). Diabetes was defined on the basis of a history of therapy with oral hypoglycemic agents or insulin at the time of inclusion. Based on the clinical information, all diabetic patients in this study were assumed to have type 2 diabetes mellitus.

We also evaluated 34 CHC patients with cirrhosis and an additional group of 12 patients with chronic hepatitis B infection. The diagnosis of liver cirrhosis was based on clinical, laboratory, and ultrasonographic findings or histological criteria. The Child-Pugh score was used to assess the severity of liver disease. Three biochemical variables [serum albumin, bilirubin, and prothrombin time (international normalized ratio, INR)] in addition to the two clinical characteristics (presence or absence of ascites and clinical signs of encephalopathy) determine the Child-Pugh score. Patients were scored as follows: 5–6 as class (group) A, 7–9 as class (group) B, and 10–15 as class (group) C. The HBV infection was confirmed by measurement of the following infection markers in the blood serum: HbsAg, HbeAg, HbcAb, HbeAb-MEIA methods, Abbotts IMX, and HBV DNA-Digene Hybrid Capture.

 The consent of the Bioethics Committee of the Wroclaw Medical University was obtained, and all patients were informed about the character of analyses made. Studies were conducted in compliance with the ethical standards formulated in the Helsinki Declaration of 1975 (revised in 1983).

### 2.2. Biochemical Analysis

 Peripheral venous blood from fasted healthy subjects and fasted CHC patients was collected in separate tubes, one containing the anticoagulant ethylenediamine tetraacetic acid and the other without serum anticoagulant. The blood was allowed to clot for 30 min at 25°C and centrifuged at 2000 × g for 15 min at room temperature, and the serum was then separated and aliquoted into tubes for storage. The tubes were stored frozen at −80°C until they were used to study different parameters.

 A wide panel of biochemical and hematological parameters were evaluated by standard automated techniques. Serum CRP levels were determined with a high-sensitivity nephelometric method using the Beckman Image Immunochemistry system (Beckman Instruments, Fullerton, CA), which has a minimum level of detection of 0.2 mg/L. The intra- and interassay coefficients of variation for measurements of CRP were 2.7% and 3.0%, respectively.

### 2.3. Determination of Tryptophan (TRP), Kynurenine (KYN), and Picolinic Acid (PA)

 The concentrations of serum TRP, KYN, and PA in patients with CHC (*n* = 51) were determined by high-performance liquid chromatography (HPLC) (22), and the content ratios of KYN to TRP (KYN/TRP) were calculated. In brief, a Waters Model 2690 (Milford, MA, USA) was used. Sample injection was controlled by a Millennium 32 data system, and a 100l loading loop was used. Tryptophan was detected with a fluorescence detector (Waters 474 scanning fluorescence detector) at an excitation wavelength of 285 nm and an emission wavelength of 360 nm; kynurenine was detected by a UV detector (Waters 2487 dual delta absorbance detector) at a wavelength of 360 nm, and picolinic acid was detected by the same UV detector at a wavelength of 265 nm. An array detector (Waters 996 photodiode array detector) was used for the identification of the peaks. To control the setup and for peak quantification, Borwin and MS Excel software was used.

 Samples were prepared as described previously [22]. Briefly, before each HPLC analysis, we used acid precipitation to dissociate the putative PA-protein complexes and to extract the samples. Every sample was spiked with methyl-DL tryptophan (as an internal standard) before the acid precipitation. Blood serum was adjusted to a final concentration of cold 2% perchloric acid and kept on ice for 30 min. Samples were centrifuged at 13 000 g for 6 min at room temperature to precipitate and separate the protein. Supernatant was neutralized by adjusting to 0.4 M potassium hydroxide, filtered through a 0.45 *μ*m filter (13 mm GHP 0.45 mm Minispike from Waters), and analyzed by HPLC. For chromatographic separation, we used the Symmetry Shield RP18 column (Waters) and the Symmetry Shield RP18 precolumn (Waters) to protect the column. After a chromatographic session was completed, once per day the system was rinsed with a gradient to/from pure methanol, and the precolumn was replaced. The identification of tryptophan catabolites was achieved by comparing the retention times and spectral data (obtained by diode-array detection) with the standard and by spiking the samples with PA, KYN, and TRP. For quantification, plasma samples were spiked with standard. Calibration curves were fitted by linear least-square regression and correlated with the concentration of methyl-DL tryptophan. Finally, the kynurenine-to-tryptophan (KYN/TRP) ratio was calculated dividing kynurenine concentrations (*μ*mol/L) by tryptophan concentrations (*μ*mol/L).

 The precision of the method was estimated by measuring the PA at four different concentrations [22]. The intraday and interday variations were determined by spiking with PA in triplicate runs 2 times within 1 day and in at least 1 run per day for 6 days. Coefficient of variation (CV) served as the indicator of the precision. Intraday and interday CV values were within 2.5%. The CV values obtained in this study are similar to those reported previously [22].

### 2.4. Statistical Analysis

 Results are expressed as median (25th percentile–75th percentile). Frequency data were compared using the *χ* ^2^ test or the Fischer's exact test when necessary. Because many of the variables analyzed did not have a normal distribution as determined by the Kolmogorov-Smirnov test, nonparametric tests were used for comparison of data. The Mann-Whitney *U* test and the Kruskal-Wallis test were used to analyze differences among two or more groups, respectively. Multiple regression analysis was conducted by a stepwise model (with *P* < 0.05 as an entrance criterion and *P* > 0.1 as a removal criterion). Variables found to significantly correlate with PA in bivariate analysis were introduced into the model as explanatory variables (WBC, hsCRP, and the presence of diabetes in all subjects or in diabetic CHC patients). Regression analysis to determine significant correlations among different parameters was performed using the Spearman correlation coefficient. Statistical significance was established at *P* < 0.05.

## 3. Results

### 3.1. TRP, KYN, and PA Serum Concentrations in Patients with Chronic Hepatitis C Infection (CHC) and Healthy Controls

**Figure 1:**
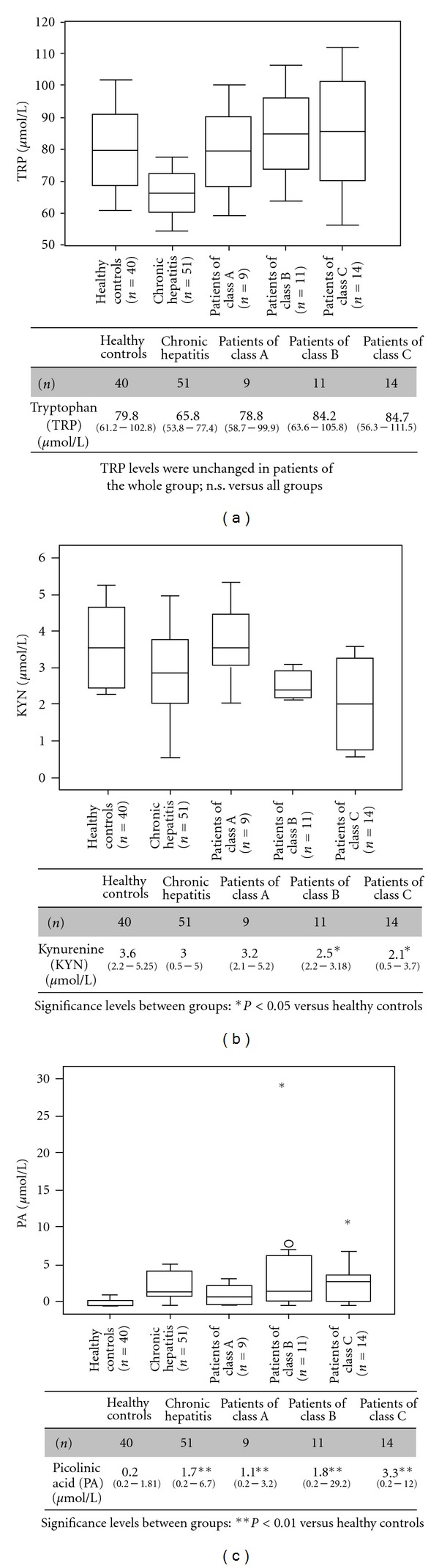
(a) Tryptophan (TRP) serum concentrations in 85 patients with chronic hepatitis C infection (CHC), according to child's stage of cirrhosis, and in a control group of 40 healthy blood donors*. P* values are given in the table. Comparisons between subgroups are illustrated with box plot graphics, where the dotted line indicates the median per group, the box represents 50% of the values, and horizontal lines show minimum and maximum values of the calculated nonoutlier values; asterisks and open circles indicate outlier values. (b) Kynurenine (KYN) serum concentrations decrease with the stage of liver cirrhosis in patients with CHC. However, in patients with Child-Pugh class A cirrhosis, the serum KYN was similar to those in healthy controls. *P* values are given in the table. (c) Picolinic acid (PA) serum concentrations increase with the stage of liver cirrhosis in patients with CHC. *P* values are given in the table.

 We analyzed 51 patients (23 males/17 females, median age 46 years, range 18–65 years) with chronic hepatitis C infection (CHC). The distribution of the stages of liver cirrhosis as defined according to the Child-Pugh score and measurements of tryptophan (TRP) and catabolites serum concentrations are presented in Figure 1. TRP and catabolites were also checked for correlations with selected biochemical markers of liver function (albumin, prothrombin ratio, and bilirubin concentration) and injury (aminotransferases).

 The concentration of TRP was measured in all 51 patients with CHC with a median of 65.8 *μ*mol/L (range 53.8–77.4 *μ*mol/L) (Figure 1(a)). In healthy controls, the serum TRP was similar to those in healthy controls in other studies [31]. Serum TRP in CHC patients without cirrhosis was only slightly reduced compared with that in other groups. However, TRP levels were unchanged in CHC patients with cirrhosis (*n* = 34) (Figure 1(a)). Unexpectedly, all CHC patients had lower kynurenine levels in their serum than healthy controls (Figure 1(b)). The kynurenine to tryptophan (KYN/TRP) ratio in CHC patients was slightly lower than that of the controls, but this difference was not statistically significant (0.044 ± 0.02 versus 0.046 ± 0.05; *P* = 0.11). Serum KYN levels were significantly lower in CHC patients with cirrhosis than those measured in healthy controls. However, in patients with Child-Pugh class A cirrhosis, the serum KYN was similar to that in healthy controls (Figure 1(b)). The relatively small number of subjects in this group is insufficient to draw a meaningful conclusion (*n* = 9). There was no significant difference in KYN levels between the Child-Pugh class A and Child-Pugh class C cirrhotic patients. The peculiarity of CHC patients is the reduction in serum TRP associated with reduced serum KYN levels. The importance of such metabolic changes in the overall clinical picture remains to be studied in detail.

 Picolinic acid (PA) serum concentrations were significantly higher in CHC patients than in healthy controls (controls median 0.2 *μ*mol/L range 0.2–1.8 *μ*mol/L, *P* < 0.01). Serum PA concentrations were higher in Child-Pugh class A to class C cirrhotic patients (*n* = 34, median 2.1 *μ*mol/L, range 0.2–29.2 *μ*mol/L) than in patients without cirrhosis (*n* = 51, median 1.7 *μ*mol/L, range 0.2–6.7 *μ*mol/L), but this difference was not statistically significant (Figure 1(c)). The levels of serum PA in all cirrhotic patients were higher than those of healthy controls, and this difference was statistically significant (*P* < 0.01) (Figure 1(c)). The levels of serum PA in patients with Child-Pugh class C cirrhosis were higher than those in Child-Pugh class A cirrhosis, but this difference was not statistically significant (*P* = 0.17) (Figure 1(c)). There was no statistically significant correlation between PA levels and the Child-Pugh score in cirrhotic patients.

 Correlation analysis of TRP, KYN, PA, and selected inflammatory markers revealed only a tendency toward an extremely weak but significant correlation between PA and hsCRP in CHC patients (*r* = 0.23, *P* < 0.05). No correlation was found between TRP, KYN, PA and biochemical markers of liver function (albumin, prothrombin ratio, and bilirubin concentration) and injury (aminotransferases). We failed to find any relation between the circulating levels of PA and TRP, KYN, or KYN/TRP ratio.

### 3.2. Association between Serum Levels of PA and Viral Etiology of Hepatitis

 Differences in serum TRP and KYN were not significant in both chronic hepatitis C and chronic hepatitis B infection. Unfortunately, efforts to conduct studies in patients with chronic hepatitis B have been hampered by the fact that the majority of chronically infected persons do not develop symptoms and therefore do not seek medical attention; consequently, the numbers included in the case series have been small. Although we could not find an association between the serum concentration of PA and viral etiology of hepatitis (Table 2), PA serum levels were considerably lower in the group with chronic hepatitis B infection (*n* = 12, median 1.02 *μ*mol/L, range 0.2–5.0 *μ*mol/L) than in the group with chronic hepatitis C infection. These results are based on a small number of subjects, so the lower PA serum levels in this group should be viewed with caution.

#### 3.2.1. Clinical and Biochemical Characteristics of Patients with Chronic Hepatitis C Infection (CHC) according to the Presence of Diabetes

The demographic, biochemical, and clinical characteristics of healthy controls and CHC patients both with and without diabetes are shown in Table 3. Distribution of sex was similar among groups. By design, glucose and HbA_1c_ levels were higher in CHC patients with diabetes than in CHC patients without diabetes, and aminotransferase levels were higher in CHC patients with and without diabetes than in the healthy controls.

PA levels in CHC patients with diabetes were higher than those in the other groups (*P* < 0.01) (Table 3). In addition, when patients with normal aminotransferases were examined, the differences remained statistically significant (data not shown). However, PA levels in nondiabetic CHC patients were similar to those in the healthy controls.

Compared with the healthy controls, CHC patients with diabetes showed a significant increase in serum concentrations of high-sensitivity C-reactive protein (hsCRP) and white blood cell (WBC) counts (Table 3). The levels of serum hsCRP in diabetic CHC patients were higher than those of the nondiabetic patients, and this difference was statistically significant (*P* < 0.01). There were no differences in platelet (PLT) counts and erythrocyte sedimentation rate (ESR) between patients and healthy controls (Table 3).

 The association study revealed only a tendency toward an extremely weak but significant correlation between PA and WBC in CHC patients with diabetes (*r* = 0.25, *P* < 0.05). However, a moderate correlation between PA levels and hsCRP was observed among the diabetic CHC patients (*r* = 0.43, *P* < 0.05).

 Using serum PA level of 3.14 *μ*mol/L as a cut-off value, the patients with CHC were divided into two groups (high or low levels of PA). The relationship between PA (high or low levels) and the other variables considered in the study in patients of the whole group (CHC patients with and without diabetes) and CHC patients without diabetes is shown in Table 4. In the whole group of patients, PA was related to the presence of diabetes and high levels of hsCRP (Table 4). However, when diabetic CHC patients were excluded, the relationship between PA and hsCRP disappeared (Table 4).

### 3.3. Multiple Regression Analysis of PA Associations

 We found that from among the variables (entered into the model, that is, WBC, hsCRP, and the presence of diabetes), only hsCRP exerted an independent effect on PA level in diabetic CHC patients. This index alone accounted for 38% of PA variance (coefficient of determination: *R* ^2^ = 0.38; multiple correlation coefficient: *R*_*m*_ = 0.62; significance of the model: *P* < 0.001). For all CHC patients, hsCRP and the presence of diabetes were found to affect PA serum level. The regression model with these 2 variables explained 60% of PA variance (*R* ^2^ = 0.60; *R*_*m*_ = 0.76, *P* < 0.001).

## 4. Discussion

 To our knowledge, this is the first report of elevated PA concentrations in patients with chronic hepatitis C infection (CHC) in which a relationship of PA to the inflammatory response has been demonstrated. Previous studies demonstrated increased levels of picolinic acid in the serum of patients with chronic liver diseases [22], but in these studies confounding factors such as age distribution, severity of liver damage (cirrhosis versus chronic hepatitis), or the presence of diabetes have not been taken into account. PA triggers cytokine-driven inflammatory reactions *in vitro*. The net effects on markers of inflammation in CHC patients are unknown.

 In the present study, we have examined the relationship between KYN pathway metabolism and inflammation by comparing both hsCRP and white blood cell count in CHC patients showing high levels of PA in the presence of low circulating KYN concentrations. After considering the confounding factors mentioned above, we provide evidence that the connection between PA and chronic inflammatory state is initiated at early stages of chronic viral hepatitis.

 Inflammation is a major component of the pathogenesis of chronic HCV infection [32, 33]. In clinical studies, significant decreases in serum TRP concentrations have been reported in patients with chronic inflammatory diseases such as HIV infection, malaria, and malignant and inflammatory bowel diseases (reviewed in [34]). Enhanced breakdown of TRP leading to the production of biologically active metabolites has been demonstrated *in vivo* under inflammatory conditions, during the rejection of transplanted tumors or in cancer patients treated with interferon-gamma (IFN-*γ*) [35], and the TRP catabolite, picolinic acid (PA), was found in the serum of patients with liver cirrhosis [22]. The present finding that PA accumulation coexists with decreased levels of TRP and the KYN/TRP ratio supports the contention that flux through the kynurenine pathway is reduced in CHC patients. The most plausible explanation for the results obtained is that downstream catabolism of quinolinic acid is reduced, thereby providing higher levels of the intermediate aminocarboxymuconate-semialdehyde (ACMS) for PA synthesis. Picolinic acid as a macrophage secondary signal causes the activation of IFN-*γ*-prime macrophage [23] and triggers cytokine-driven inflammatory reactions *in vitro*. On the other hand, the recruitment of circulating leukocytes to an inflammatory site in response to stimuli such as the hepatitis C virus is a crucial step in the development of chronic inflammatory responses. In these conditions, extravasation of circulating leukocytes requires communication with vascular endothelial cells that in turn depends on an interrelated network of events involving the proinflammatory cytokines (i.e., interleukin [IL]-1*β*, IL-6, and tumor necrosis factor [TNF]-*α*), adhesion molecules, and chemokines (notably MIP-1-*α* and MIP-1-*β*). The experiments in cell showed that PA is an activator of the inflammatory chemokines macrophage-inflammatory-protein- (MIP-) 1-*α* and MIP-1-*β* mRNA expression macrophages through *a novo* protein synthesis-dependent process [24]. Thus, the very weak correlation between PA levels and inflammatory marker C-reactive protein (hsCRP) may suggest an indirect contribution of PA to the maintenance of the inflammatory milieu through other mediators. Finally, the values of PA in CHC patients and normal aminotransferases suggest that PA might have a role in the early stages of chronic viral hepatitis by amplifying the chronic inflammatory state already caused by the hepatitis C virus [32, 33].

 The association between tryptophan content and diabetes is supported by the observation that tryptophan levels are reduced in age-related metabolic disorders [36]. The specific mechanisms that could lead to PA enhancement in CHC patients with diabetes remain to be explained. It is possible to speculate that elevated levels of PA may occur as a result of increased activity of the enzyme responsible for the synthesis of PA. ACMSD (picolinate carboxylase, 2-amino 3-carboxymuconate 6-semialdehyde (ACMS) decarboxylase; ACMSD) is a key enzyme directing kynurenine pathway metabolism towards PA production and is mainly present in liver, kidney, and brain tissue. One study showed that in liver of streptozotocin-induced diabetic rats, hepatic ACMSD activity is increased markedly, and insulin injection suppresses this elevation [37]. These studies demonstrated that changes in ACMSD activity are reflected by serum PA levels. Finally, our results showed that diabetes but not HCV infection was independently related to PA in patients with chronic hepatitis C infection.

 Accumulating evidence suggests that chronic hepatitis C virus infection increases the risk of atherosclerotic cardiovascular disease by causing insulin resistance and diabetes [1, 3, 38]. Some research has suggested that hepatitis C virus may also trigger the release of proinflammatory cytokines such as TNF-*α*, IL-6, IL-1*β*, and inflammatory CRP [39, 40]. The results of general population-based studies have also demonstrated a relationship between hepatitis C virus persistence and carotid atherosclerotic plaques and intima-media thickness [41]. Interestingly, this recent research suggests that active macrophage infiltration into the arterial wall induces the development of an early atherosclerotic lesion [42]. On the other hand, as we mentioned above, PA is capable of activating macrophages and inducing the expression of the inflammatory chemokines MIP-1*α* and MIP-1*β* belong to the *β*-chemokines family, which plays a key role in the recruitment of mononuclear cells (monocytes and T lymphocytes) into atherosclerotic lesions. Together, our observations show that the simultaneous elevation of PA and hsCRP levels in CHC patients with diabetes suggest that their local tissue production is linked. This is in accordance with experimental data showing overexpression in plaques of both *β*-chemokines [43] as well as CRP [44] and may be due to global activation of cells that synthesize and secrete these molecules. Even if the correlation between PA and WBC was extremely weak, we demonstrated clear association with inflammatory CRP. Finally, our results show that the elevation of PA was dependent on inflammation status because in diabetic CHC patients with normal hsCRP concentrations, PA level was not significantly elevated in comparison with healthy controls (data not shown). Although CRP can merely be a marker of an underlying inflammatory process, it is possible that CRP may be directly involved in the pathogenesis of inflammation/atherosclerosis [45]. In this context, the association between picolinic acid and the presence of inflammation in CHC patients with diabetes could represent one of the mechanisms involved in atherosclerotic process in this population. However, the mechanisms underlying this association are still not completely clarified.

 Some limitations of the current study should be considered. Firstly, the small set of 51 patients included in this study may have resulted in limited power to discriminate between categories and levels of exposure. Secondly, the part of our patients, categorized as “CHC patients without diabetes” may have diabetes without symptoms, and many of them may develop diabetes in the future. Finally, because of its cross-sectional nature, this study is limited in terms of deriving mechanistic conclusions; however, results of this study are hypothesis generating and provide insights into the relationship between PA levels and inflammatory response and the presence of diabetes in patients with CHC.

 In conclusion, we have documented significant elevation in serum levels of PA in patients with CHC. In addition, circulating PA was associated with inflammatory marker CRP and the presence of diabetes, but not with markers of liver injury aminotransferases. In summary, our results provide further evidence for the presence of high levels of hsCRP in CHC patients with diabetes, and we propose a role for PA in this context. The results of our study should be interpreted as trends to be confirmed by further studies with larger sample size.

## Figures and Tables

**Table 1:** Clinical and biochemical characteristics of patients with chronic hepatitis C infection (CHC) and healthy controls.

	Healthy Controls	CHC patients	Cirrhotic CHC patients
(*n*)	40	51	34
Male : female ratio	23 : 17	22 : 29	14 : 10
Age (years)	32.5 (19–65)	46* (18–65)	43 (26–70)
BMI (kg/m^2^)	28 (22–33)	25.6 (22–31)	—
Diabetes mellitus (%)	—	43%	—
ALT (U/L)	24 (20–28)	93**(10–392)	47** (16–79)
AST (U/L)	27 (23–30)	68**(12–272)	79** (19–150)
*γ*-GT (U/L)	26 (25–28)	62.5* (9.0–321)	92** (78–106)
Total cholesterol (mg/dL)	188 (140–200)	160 (154–209)	140* (130–203)
LDL cholesterol (mg/dL)	116 (80–130)	110 (78–141)	92 (80–135)
HDL cholesterol (mg/dL)	60 (35–80)	43 (30–52)	40 (30–75)
Triglycerides (mg/dL)	76 (35–150)	84 (76–183)	78 (30–190)
Albumin (g/L)	45 (36–57)	41 (39–48)	30* (16–45)
hsCRP (mg/L)	1.2 (0.1–4.8)	3.5*(0.22–4.45)	4.9** (3.5–10.5)

Data reported as median (range). Statistical significance: * *P* < 0.05; ** *P* < 0.01 versus healthy controls. AST, aspartate aminotransferase; ALT, alanine aminotransferase; *γ*GT, gamma glutamyltransferase; hsCRP, high-sensitivity C-reactive protein.

**Table 2:** Serum tryptophan (TRP), kynurenine (KYN), and picolinic acid (PA) in patients with chronic viral hepatitis.

	TRP (*μ*mol/L)	KYN (*μ*mol/L)	PA (*μ*mol/L)
Healthy controls (*n* = 40)	79.8 (61.2–102.8)	3.6 (2.2–5.25)	0.2 (0.2–1.81)
Chronic hepatitis C infection (*n* = 51)	65.8 (53.8–77.4)	3.0 (0.5–5.0)	1.7*(0.2–6.7)
Chronic hepatitis B infection (*n* = 12)	61.5 (48.4–74.2)	2.6 (0.44–4.1)	1.02*(0.2–5.0)

Significance levels between groups: * *P* < 0.05 versus healthy controls.

**Table 3:** Clinical and biochemical characteristics of patients with chronic hepatitis C infection (CHC) according to the presence of diabetes.

	Healthy controls	CHC patients with diabetes	CHC patients without diabetes
(*n*)	40	22	29
Male : female ratio	23 : 17	10 : 12	12 : 17
Age (years)	32 (19–65)	46 (21–65)	45 (18–60)
BMI (kg/m^2^)	28 (22–33)	27 (23–31)	25.8 (22–29)
Glucose (mmol/L)	4.6 (4.0–5.2)	7.6^∗∗+^ (5.7–9.5)	5.2 (4.7–5.7)
HbA_1c_(%)	—	7.8^+^ (5.0–9.8)	5.4 (5.0–6.0)
ALT (U/L)	24 (20–28)	86** (10–392)	74* (20–144)
AST (U/L)	27 (23–30)	68* (12–272)	53* (20–97)
Albumin (g/L)	45 (36–57)	43 (39–44)	45 (43–48)
PA (*μ*mol/L)	0.2 (0.2–1.81)	1.9^∗∗+^(0.8–6.7)	0.32 (0.2–1.5)
hsCRP (mg/L)	1.2 (0.1–4.8)	3.14^∗+^(0.36–4.45)	1.51 (0.22–1.9)
ESR (mm/h)	6 (5–7)	8 (3.5–22)	6 (5–7)
PLT (× 10^9^/L)	200 (150–220)	205 (158–240)	207 (161–242)
WBC (× 10^9^/L)	4.6 (4.2–5.1)	6.7*(5.1–8.8)	5.3 (4.5–7.4)

Data reported as median (range). Significance levels between groups: * *P* < 0.05; ** *P* < 0.01 versus healthy controls; ^+^ *P* < 0.01 versus CHC patients without diabetes. ALT, alanine aminotransferase; AST, aspartate aminotransferase; CRP, C-reactive protein; ESR, erythrocyte sedimentation rate; PA, picolinic acid; PLT, platelet; WBC, white blood cells.

**Table 4:** Comparison between the patients of the whole group (CHC patients with and without diabetes) and nondiabetes CHC patients classified according to the finding of low (≤3.14 *μ*mol/L) and high (>3.14 *μ*mol/L) levels of picolinic acid (PA).

****	CHC patients (*n* = 51)		CHC patients without diabetes (*n* = 29)	
	PA ≤ 3.14 *μ*mol/L	PA > 3.14 *μ*mol/L	PA ≤ 3.14 *μ*mol/L	PA > 3.14 *μ*mol/L
*n* (%)	28 (54)	23 (46)	16 (56)	13 (44)
Age (years)	40 (18–52)	42 (26–65)	42 (21–60)	43 (18–58)
BMI (kg/m^2^)	24.9 (22–31)	25.7 (23–27)	24.8 (22–28)	26 (23–29)
hsCRP (mg/L)	2.4 (0.51–2.9)	3.7^+^ (0.22–4.45)	0.88 (0.25–1.9)	1.0 (0.22–1.75)
HbA_1c_(%)	5.8 (5.0–7.9)	7.0^++^ (5.4–9.8)	—	—
Diabetes *n* (%)	5 (10)	17 (33)^ ++^	—	—
Total cholesterol (mg/dL)	165 (154–198)	178.2 (161–209)	173.8 (161–196)	168.5 (164–209)
LDL cholesterol (mg/dL)	84 (78–102)	86 (80–141)	102 (96–139)	98 (95–141)
HDL cholesterol (mg/dL)	37 (33–49)	36 (30–52)	37 (33.0–49.5)	38.6 (34–52)
Triglycerides (mg/dL)	85 (76–150)	87 (80–183)	84 (76–148)	81 (79–137)
ALT (U/L)	43 (10–319)	57 (10–392)	42 (20–137)	53.7 (20–144)
AST (U/L)	35 (12–143)	45 (16–272)	33.5 (20–87)	35 (20–97)
*γ*-GT (U/L)	29 (9.0–101)	34 (12–321)	28.7 (10.0–99.7)	32.7 (14–309)

Significance between groups: ^+^ *P* < 0.05; ^++^ *P* < 0.01 by multivariate analysis. AST, aspartate aminotransferase; ALT, alanine aminotransferase; *γ*GT, gamma glutamyltransferase; hsCRP, high-sensitivity C-reactive protein.
